# Proteomic analysis of maize grain development using iTRAQ reveals temporal programs of diverse metabolic processes

**DOI:** 10.1186/s12870-016-0878-1

**Published:** 2016-11-04

**Authors:** Tao Yu, Geng Li, Shuting Dong, Peng Liu, Jiwang Zhang, Bin Zhao

**Affiliations:** State Key Laboratory of Crop Biology and College of Agronomy, Shandong Agricultural University, Taian, 271018 Shandong People’s Republic of China

**Keywords:** Maize, Grain development, Proteomics, iTRAQ, Starch

## Abstract

**Background:**

Grain development in maize is an essential process in the plant’s life cycle and is vital for use of the plant as a crop for animals and humans. However, little is known regarding the protein regulatory networks that control grain development. Here, isobaric tag for relative and absolute quantification (iTRAQ) technology was used to analyze temporal changes in protein expression during maize grain development.

**Results:**

Maize grain proteins and changes in protein expression at eight developmental stages from 3 to 50 d after pollination (DAP) were performed using iTRAQ-based proteomics. Overall, 4751 proteins were identified; 2639 of these were quantified and 1235 showed at least 1.5-fold changes in expression levels at different developmental stages and were identified as differentially expressed proteins (DEPs). The DEPs were involved in different cellular and metabolic processes with a preferential distribution to protein synthesis/destination and metabolism categories. A K-means clustering analysis revealed coordinated protein expression associated with different functional categories/subcategories at different development stages.

**Conclusions:**

Our results revealed developing maize grain display different proteomic characteristics at distinct stages, such as numerous DEPs for cell growth/division were highly expressed during early stages, whereas those for starch biosynthesis and defense/stress accumulated in middle and late stages, respectively. We also observed coordinated expression of multiple proteins of the antioxidant system, which are essential for the maintenance of reactive oxygen species (ROS) homeostasis during grain development. Particularly, some DEPs, such as zinc metallothionein class II, pyruvate orthophosphate dikinase (PPDK) and 14-3-3 proteins, undergo major changes in expression at specific developmental stages, suggesting their roles in maize grain development. These results provide a valuable resource for analyzing protein function on a global scale and also provide new insights into the potential protein regulatory networks that control grain yield and quality.

**Electronic supplementary material:**

The online version of this article (doi:10.1186/s12870-016-0878-1) contains supplementary material, which is available to authorized users.

## Background

The grains of cereal crops have high agronomic value; this is particularly true of maize (*Zea mays* L.), which is cultivated worldwide and is one of the most important crops as a source of food, animal feed, and renewable resources. Improvement of the yield and quality of grain is a major objective of maize breeding. Molecular biology technologies and genomics have received increasing attention for maize breeding as they provide new and more efficient selection criteria [1]. Therefore, a better understanding of the metabolic processes and underlying molecular mechanisms associated with grain development will provide new insights that will enable future increases in grain yield and quality.

Over the past several decades, much progress has been made in understanding maize grain development, which is initiated by a double fertilization process and is divided into three main stages: the lag, grain filling, and maturation stages [2, 3]. The lag stage encompasses events up to 12 d after pollination (DAP) and is characterized by a rapid expansion in cell number and sizes; this increase determines the size of the sink for the subsequent accumulation of storage molecules. The grain filling stage lasts from 12 to 40 DAP, and is characterized by the onset of synthesis and accumulation of storage molecules. During this stage, starch, which is composed of amylose and amylopectin, is the major stored component and is synthesized from imported sucrose. Various enzymes synthesize starch and then trim and pack the molecules as semi-crystalline starch granules in amyloplasts [4–6]. The maturation stage occurs from 40 to 70 DAP, and is characterized by dehydration of the grains, which gradually go into a quiescent dormancy state. The duration of each stage varies depending on genetic background, environmental, and cultural conditions [7]. Although our understanding of the morphological and physiological changes during grain development has increased, the underlying molecular regulatory mechanisms are still largely unknown [8–10].

The identification of gene activities and functions is an effective method for exploring molecular regulatory mechanisms. Large-scale genome-wide expression analyses using microarrays, cDNA libraries, and RNA sequencing (RNA-seq) have described large numbers of genes that are preferentially expressed in embryogenesis or accumulation of storage compounds during maize grain development [1, 9–13]. For example, a dynamic transcriptomics analysis using an RNA-seq strategy in maize embryo, endosperm, and whole grain from fertilization to maturity identified 26,105 genes involved in programming grain development; moreover, 1258 of these genes were determined to be grain-specific [10]. Although information has been reported on the genes involved in grain development, there is a lack of equivalent detail at the protein level despite their role as direct regulators of cell activity. More importantly, transcription patterns are not always directly associated with the expression of the corresponding protein, as has been shown in maize [14], rice [15], cotton [16], and *Arabidopsis* [17]. Therefore, direct proteomics research is also essential for monitoring grain developmental profiles.

To date, the reported proteomic studies of grain development have mainly used two-dimensional gel electrophoresis (2-DE). Such studies have been performed in many species, including rice [15, 18, 19], wheat [20], *Arabidopsis* [17], barley [21], castor [22], *Medicago truncatula* [23], and soybean [24, 25]. However, some types of protein can’t be analyzed by 2-DE as it has the inherent restrictions of being unable to separate hydrophobic proteins, low identification rate, and lack of accurate quantitative information [26, 27]. Recently, an alternative approach has been developed using isobaric tag for relative and absolute quantitation (iTRAQ) as a mass spectrometry-based quantitative technology; this technique overcomes some of the limitations of 2-DE, especially for multiple samples, and allows identification of a greater number of proteins to provide more reliable quantitative information [28, 29]. The advantages of iTRAQ technology have been exploited to identify and quantify 2165 proteins in developing rice embryos [30] and 1815 proteins in wheat grains [31].

In the past, several proteomics analysis of maize whole grain or embryo and endosperm has been carried out. Based on 2-DE, Méchin et al. [32] established a proteome reference map for maize endosperm, and 504 proteins were identified that were mainly assigned to the metabolic and protein destination category. They subsequently quantified 409 proteins at seven development stages between 4 and 40 DAP and showed that the dynamic expression patterns of these proteins are consistent with the important developmental shift from cell growth and differentiation to storage [8]. In order to explore the regulatory factors which are critical for maize grain filling, Jin et al. [7] found 39 proteins in endosperm and 43 proteins in embryo, which were differentially expressed in three elite maize hybrids during the linear filling phase (17–28 DAP), by using 2-DE, and the further functional analysis revealed that proteins related to glycolysis and redox homeostasis were emphasized in the endosperm, while proteins involved in fatty acid biosynthesis were emphasized in the embryo. 40 proteins were also found to be differentially expressed after grain ageing by 2-DE, indicating that artificial ageing affected the proteome of the dry maize grains [33]. In other studies using 2-DE, the expression level of proteins related to maize embryo desiccation tolerance was studied [34] and grain viability was investigated [35]. However, because of the limitations of the 2-DE method, these studies could only study a relatively small number of proteins. In a recent study, using mass spectrometry, Walley et al. [14] mapped an atlas of proteotypes for the developing maize grain based on protein abundance and levels of protein phosphorylation. As a result, 14,165 proteins and 18,405 phosphopeptides (from 4511 proteins) were quantified in different grain compartments and development stages, including embryo (20, 38 DAP and 2 d after imbibition), endosperm (8, 10, 12 and 27 DAP) and aleurone/pericarp (27 DAP), and this study further revealed that many of the most abundant proteins were not associated with detectable levels of their mRNAs [14]. The atlas provided rich resources for identify kinase-substrate relationships as well as the reconstruction of biochemical and signaling networks that underpin grain development and grain storage product production. Although considerable work of proteomics investigation in maize grain has been performed, these studies mainly focused on maize grain different components (embryo, endosperm and aleurone/pericarp) and several time points. Meanwhile, maize had a larger genome and a more complex proteome than model plants such as *Arabidopsis* and rice, the regulatory mechanisms that are important for maize grain development still require further study. Importantly, to our knowledge, a systematic proteomics analysis of the entire development process in maize grain based on iTRAQ has not been reported. Therefore, we analyzed the dynamic changes in protein expression in maize grain at eight sequential developmental stages from 3 to 50 DAP, a period that covers three major development processes using iTRAQ technology. Our results revealed a global shift of protein expression patterns corresponding to grain development, which serve as a valuable resource for analyzing protein function on a global scale and providing new insights into the potential protein regulatory networks that control grain yield and quality.

## Results and discussion

### Physiology characteristics of maize grain at eight developmental stages

**Fig. 1:**
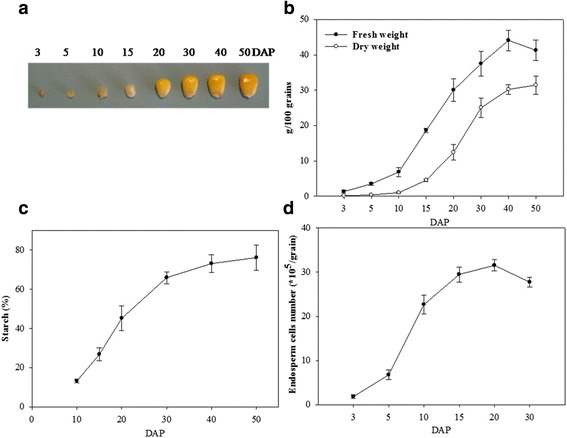
Development of maize grains during the experimental period. **a** Maize grains at the eight stages of development. **b** Changes in fresh and dry weights of developing grains. At least 100 grains were analyzed at each stage. **c** Changes in total starch contents of developing grains. Values are expressed as a percentage of grain dry weights. **d** Dynamic changes of endosperm cell number in grains. Error bars represent SD of three replicates

Whole maize grains were sampled at 3, 5, 10, 15, 20, 30, 40, and 50 DAP (Fig. 1a), and the characteristics of the developing grains were recorded at each sampling day (Fig. 1b–d). During grain development, both fresh and dry grain weight slowly increased from 3 to 10 DAP, followed by a more rapid increase to 40 DAP (Fig. 1b). After 40 DAP, dry weight continued to increase until 50 DAP, whereas fresh weight declined, indicating that the developing grains had entered the desiccation stage after 40 DAP. Total starch content increased rapidly between 10 and 30 DAP, and then more slowly until 50 DAP (Fig. 1c), indicating that the period 10–30 DAP was the key stage for grain starch synthesis and accumulation. In addition, analysis of endosperm proliferation showed that the number of endosperm cells increased rapidly from 3 to 10 DAP, and then more slowly to reach a maximum at 20 DAP (Fig. 1d). Overall, our results suggest that up to 10 DAP developing grains mainly undergo active cell division and differentiation, followed by grain filling until 40 DAP, and then enter into the desiccation stage after 40 DAP. Therefore, these three periods are approximately representative of the three main stages of maize grain development, i.e., early (3–10 DAP), middle (10–40 DAP), and late (40–50 DAP).

### Identification and relative quantitation of proteins in maize grain

**Fig. 2:**
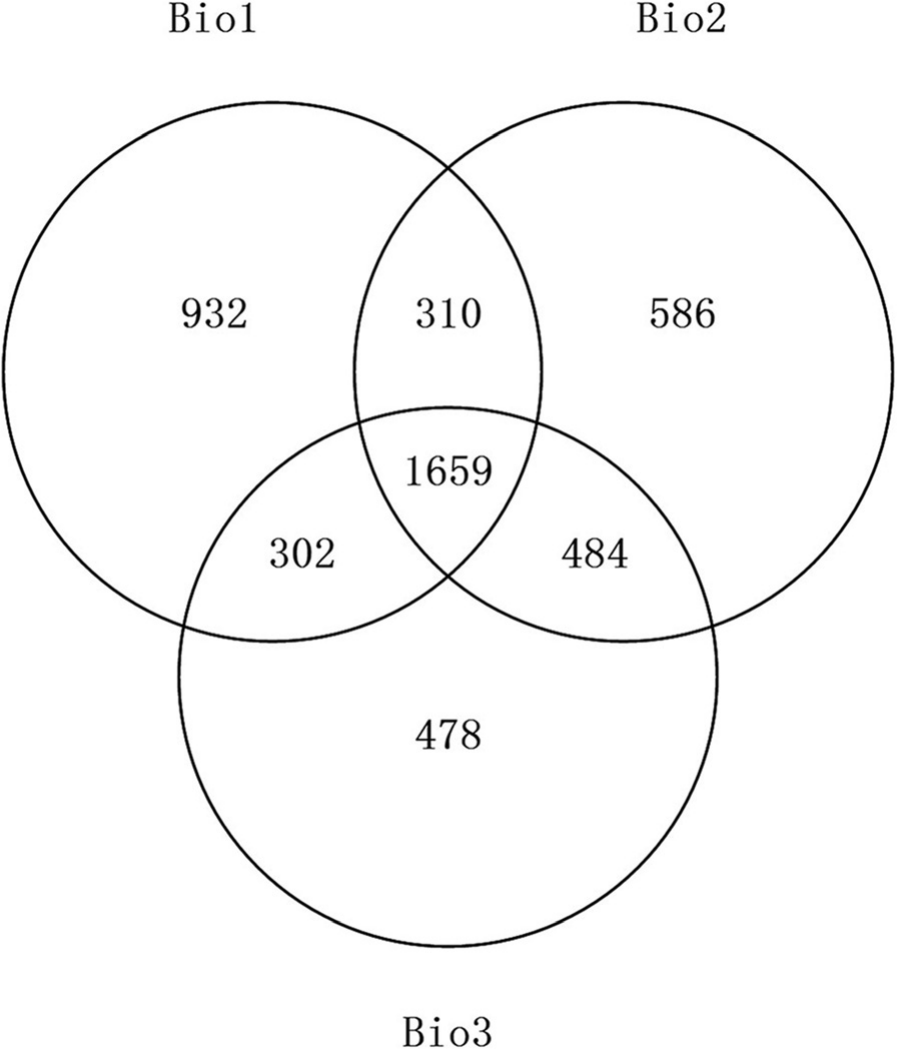
Venn diagram representing the overlap of the identified proteins in the three biological repeats. The Bio1, Bio2, Bio3 represent the first, second and third biological replicates, respectively

**Fig. 3:**
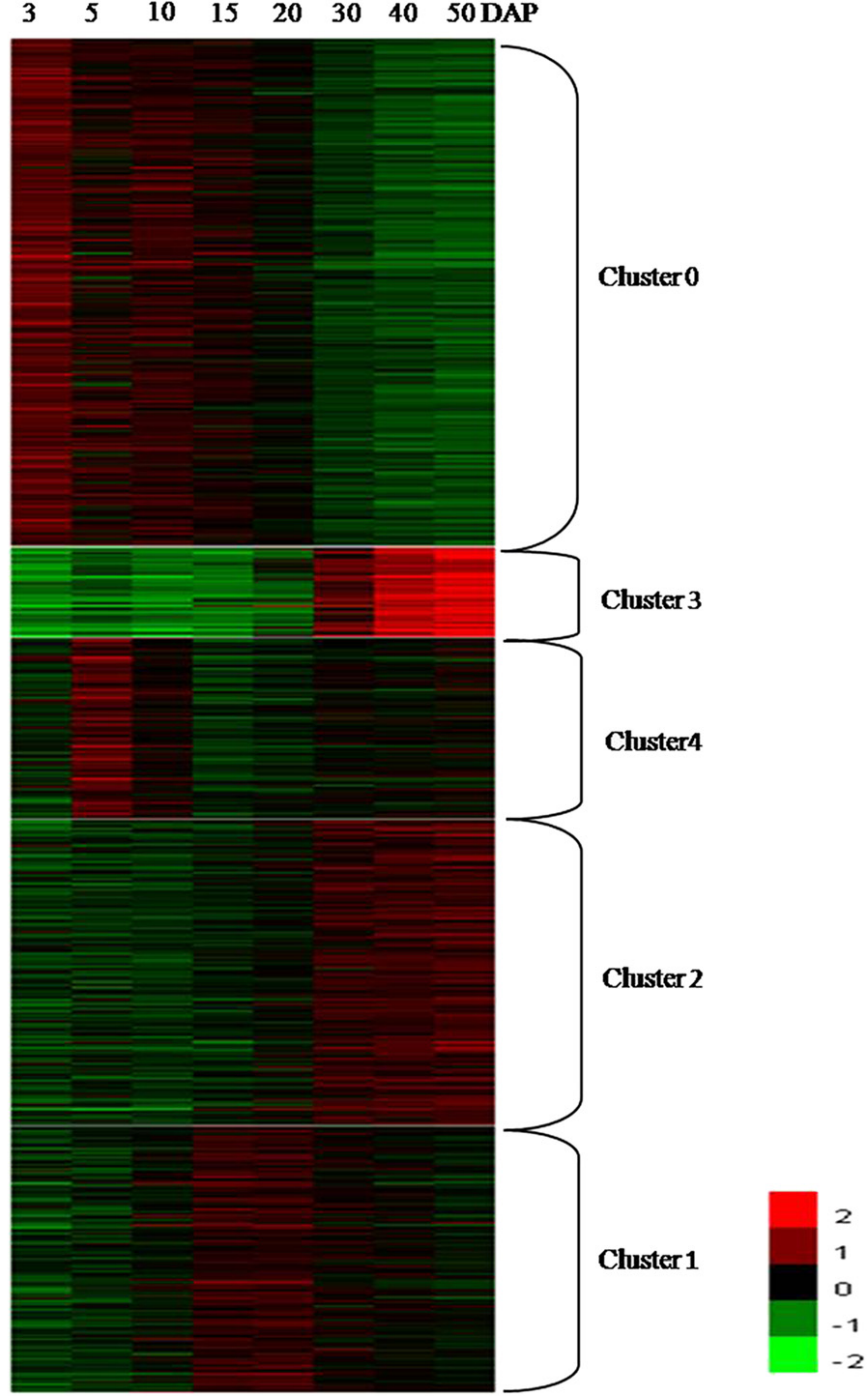
K-means clustering of functional DEPs at the eight developmental stages. The functional DEPs are listed in Additional file 4, with information on their cluster assignment

The analysis of total proteins and of changes in protein expression was performed using iTRAQ-based proteomics with three biological replicates. Strict identification and quality criteria were also used (for details, see “Methods”). The analysis identified 4751 proteins in maize grain, of which 2755 proteins were common in two biological replicates (Fig. 2; Additional file 1); 2639 of these proteins were quantified (Additional file 2). The number of identified and quantified proteins was greater than that from previous proteomic analyses using 2-DE [8, 32], clearly demonstrating that iTRAQ technology has greater potential for protein identification and quantification compared to conventional gel-based methods. Simultaneously, compared to a recent proteomics analysis that had quantified 14,165 proteins and 18,405 phosphopeptides (from 4511 proteins) in the developing maize embryo (20, 38 DAP and 2 d after imbibition), endosperm (8, 10, 12 and 27 DAP) and aleurone/pericarp (27 DAP) by mass spectrometry [14], the number of identified and quantified proteins was still relatively small in our study. However, our study described the dynamic changes of proteome in maize grain during entire development stages. A 1.5-fold cut-off change in expression (p ≤ 0.05) during development was used to identify significant changes in the abundance of differentially expressed proteins (DEPs). A total of 1235 proteins were classified as DEPs and K-means clustering analysis assigned these proteins to five expression cluster groups (c0, c1, c2, c3, and c4; Table 1; Fig. 3). The largest cluster was c0, with 466 proteins in this group; expression of these proteins gradually declined from 3 to 50 DAP. The next largest clusters were c2 (279 proteins) and c1 (243 proteins). The level of expression of c2 proteins increased gradually at 30 DAP and reached a maximum at 50 DAP. By contrast, c1 proteins showed considerable accumulation at 15–20 DAP, and occasionally to 30 DAP, but decreased thereafter. Cluster c3 consisted of 82 proteins whose expression patterns were similar to those of c2 proteins except that they showed a large increased from 30 to 50 DAP. Cluster c4 consisted of 165 proteins and their expression contrasted that of c1 proteins by having peaks at 6 and 50 DAP. These results suggested that different patterns of protein regulation were correlated with early, middle, and late stages of grain development.

**Table 1:** K-means clusters of DEPs and distribution of proteins involved in each category or subcategory in different clusters

Categories or Subcategories	c0	c1	c2	c3	c4	Total
01 Metabolism	84	95	32	7	29	247
01.01 Carbohydrate metabolism	5	7	9	1	10	32
01.02 Starch synthesis	2	17	3	0	1	23
01.03 Glycolysis	4	15	3	0	2	24
01.04 Pyruvate dehydrogenase complex	1	3	1	0	0	5
01.05 TCA pathway	4	2	2	0	0	8
01.06 Alcoholic fermentation	2	5	0	0	0	7
01.07 Pentose phosphate pathway	6	1	0	0	0	7
01.08 Amino acid metabolism	18	22	5	0	3	48
01.09 Nucleotides metabolism	6	7	2	0	2	17
01.10 Lipid and sterol metabolism	23	9	5	6	7	50
01.11 Secondary metabolism	13	7	2	0	4	26
02 Protein synthesis and destination	124	63	82	15	27	311
02.01 Protein synthesis	56	21	24	0	13	114
02.02 Protein folding and modification	23	18	26	3	7	77
02.03 Proteolysis	20	13	21	2	4	60
02.04 Storage protein	2	3	6	10	0	21
02.05 Protein transport	23	8	5	0	3	39
03 Cell growth/division	75	11	13	1	13	113
03.01 Cell growth and DNA related	60	10	10	0	8	88
03.02 Cell wall related	15	1	3	1	5	25
04 ROS homeostasis	14	12	15	2	15	58
05 Defense/stress response	14	7	35	41	8	105
06 Signal transduction	16	9	13	1	7	46
07 Transporters	50	5	17	1	15	88
08 Transcription	18	3	23	1	10	55
09 Photosynthesis	1	1	0	0	6	8
10 PPDK	0	2	0	0	0	2
11 14-3-3 protein	3	0	0	0	0	3
12 Unclassified	67	35	49	12	36	199

The clusters (c0 to c4) were created by Gene Cluster 3.0; raw data for the clusters are listed in Additional file 4

**Fig. 4:**
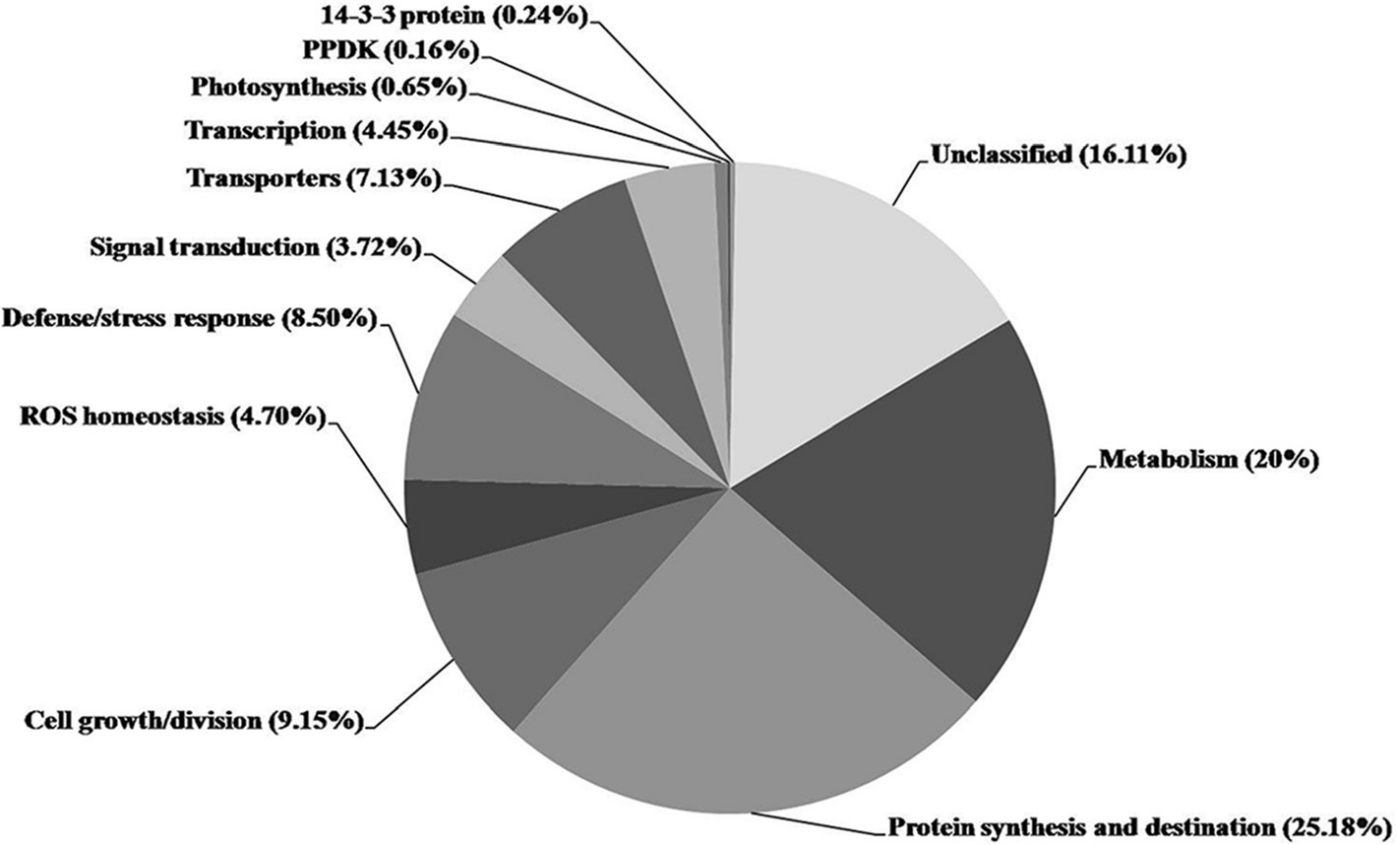
The functional distribution of DEPs identified from developing maize grains

Among the 1235 DEPs, 572 were annotated as uncharacterized proteins. To obtain functional information on these proteins, we performed a BLAST analysis to search for homologous proteins; this search identified homologous sequences in other species for 437 of the uncharacterized protein sequences (Additional file 3). According to the presumed biological function listed in UniProt and the scheme for functional category classification for maize endosperm [8] and rice grain [15] proteins, the 1235 DEPs were classified into different functional categories. Proteins involved in protein synthesis/destination and metabolism comprised the largest groups, approximately 25.18 and 20 %, respectively (Fig. 4), suggesting the functional importance of metabolism and protein synthesis/destination during grain development. In order to obtain more detailed information about these two functional categories, DEPs involved in protein synthesis/destination and metabolism were further assigned to 5 and 11 subcategories, respectively (Table 1).

### Protein expression characteristics during grain lag stage

The grain lag stage (0–10 DAP) was characterized by active cell division and enlargement to increase the grain sink size for subsequent accumulation of storage material. Previous proteomic analysis had showed that cytoskeleton proteins (actin and tubulin) which play crucial roles in cell division and enlargement during embryogenesis [36], were accumulated to highest levels at this stage. In this study, we confirmed that most DEPs related to cell growth/division (75 of 113 proteins) preferentially accumulated at this stage (c0; Table 1), not only including the cytoskeleton proteins but also other proteins, such as proliferating cell nuclear antigen and histone (Additional file 4). Proliferating cell nuclear antigen is involved in DNA repair and cell cycle regulation [37]. Meanwhile, the formation of cell wall and unit membranes (the structural components of cells) is also enhanced [38, 39]. As a result, most of the DEPs associated with cell wall formation (15 of 25 proteins), such as UDP-glucose 6-dehydrogenases (B6T9P0 and B6TBY8) and the xyloglucan endotransglucosylase/hydrolase (B4G1Z2) showed maximal accumulation at this early stage (Additional file 4). The UDP-glucose 6-dehydrogenases are involved in cell wall polysaccharide synthesis, while xyloglucan endotransglucosylase/hydrolase functions in loosening and extending the cell wall [38]. For the formation of cell membranes, approximately half of the lipid/sterol metabolism-related proteins (23 of 50 proteins) showed enhanced accumulation, similar to that found for cell wall-associated proteins (c0; Table 1); among the enhanced lipid/sterol metabolism-related proteins was as saposin-like type B protein (B6T780), which can interact with lipids [40] and may function as a surfactant to reduce surface tension [41]. It will be of interest to fully elucidate the role of this protein in reducing the cell surface tension that results from cell expansion at this early stage of maize grain development.

Active protein turnover was found during early development of rice grain [15]. Here, our analysis showed that one-third of DEPs involved in proteolysis (20 of 60 proteins) showed maximal accumulation at the early stage (c0; Table 1). Some of these DEPs were key components of the ubiquitin/26S proteasome pathway (9 of 20 proteins), an important protein degradation pathway for diverse cellular and developmental events [42, 43]. Meanwhile, a large number of DEPs related to protein synthesis (56 of 114 proteins) and protein transport (23 of 39 proteins) showed similar accumulation patterns to those of proteolysis-related proteins (c0; Table 1). Overall, these results suggested that protein turnover and rearrangements were also important for maize grain cell division and enlargement at the early stage.

In maize, proteomics analysis about developing grain had revealed the regulation of glycolysis and tricarboxylic acid (TCA) cycle at protein levels [7, 8]. The significantly decreased expression of proteins involved in these two pathways marks that the grain are entering mature stage, which indicates the importance of the regulation of glycolysis and TCA cycle for grain development. However, the regulation of pentose phosphate pathway (PPP) as another important respiratory pathway in maize grain is relatively little known. The PPP is central to plant metabolism and functions in providing reducing power and pentose phosphates for multiple metabolic pathways [44]. The reducing power is produced in the oxidative part of the PPP (oxPPP) by glucose-6-phosphate 1-dehydrogenase (G6PDH) and 6-phosphogluconate dehydrogenase (6PGDH); G6PDH is considered to be rate-limiting for oxPPP [44, 45]. Our analysis identified seven DEPs related to PPP, including two G6PDHs (B6TSB3 and C0PFX0) and three 6PGDHs (A0A096SF47, Q9SBJ3 and B4FSV6); these two types of PPP proteins have not been identified in previous proteomics investigations of maize grain [8, 34, 35]. Surprisingly, these PPP protein types preferentially accumulated at the early stage (Table 1; Additional file 4), suggesting that oxPPP is highly active. G6PDH has been found to show a similar pattern of decreasing expression during castor grain development [22]. Overall, these results suggested that active PPP was crucial for maize early grain development, possibly providing reducing power and pentose phosphates for the fatty acid and nucleic acid synthesis required for membrane synthesis and cell division at this stage [44].

### Grain filling in the transition from cell growth to starch synthesis and accumulation

At the mid stage of grain development (10–40 DAP), grains showed a small increase in cell numbers and size, whereas storage materials (mainly starch) began to be rapidly synthesized and accumulated. A striking observation is that DEPs related to cell growth were rapidly down-regulated compared to the early stage, while starch synthesis related proteins reached their maximal levels at the mid stage (Table 1), reflecting the transition from cell division and differentiation to grain filling. In previous studies, the proteomic analysis of the expression changes of proteins related to starch synthesis during maize grain development had suffered many restrictions because a small number of these proteins were detected in grain [7, 8] and quantitative information of these proteins didn’t cover the entire grain development stages [14]. In contrast, the iTRAQ method identified and quantized a considerable number of key proteins related to starch biosynthesis, including sucrose synthase (SuSy), ADP-glucose pyrophosphorylase (AGPase), ADP-glucose brittle-1 transporter (BT1), starch synthase (SS), starch branching enzyme IIb (SBEIIb), isoamylase I (ISAI), and starch phosphorylase (SP). Most of these proteins were grouped into the c1 cluster (Table 1); this analysis provides a comprehensive view of starch biosynthesis during maize grain development.

1. SuSy, AGPase, and BT1
  In plant sink organs, the primary mobilization of sucrose for starch synthesis is regulated by SuSy [46]; AGPase catalyzes the first key regulatory step in starch synthesis by converting glucose-1-phosphate (G1P) into ADP-glucose (ADP-Glu) [5]. In maize grain, SuSy transcript levels were reported to increase until the middle of development and to decline thereafter [10]. In our study, four isozymes of SuSy were identified as DEPs (Fig. 5). One SuSy isozyme (C0P6F8) exhibited a stable expression level until 20 DAP, and rapidly decreased thereafter. Two other isozymes (Q93WS3 and B6U1D7) increased until 20 DAP and one (K7VDR8) until 30 DAP, and then all decreased; these patterns are consistent with those of transcription. This suggests that two types of SuSy may be active during the early (type I) and middle (type II) stages of grain development. Five isoforms of AGPase were identified as DEPs (Fig. 5), and the expression level of four of these peaked around 20 DAP, when starch synthesis was at its greatest. In cereal grain such as maize and rice, cytoplasmic AGPases contribute most of the total AGPase activity [5]. Much of the ADP-Glu used for the biosynthesis of starch is synthesized in the cytosol and then imported into the amyloplast by BT1, the activity of which is closely related to the transport efficiency of ADP-Glu [47, 48]. One BT1 was identified in our study (Fig. 5), and peaked in level around 20 DAP, similar to the expression pattern of AGPase. The co-expression of AGPase and BT1 may ensure the efficient supply of ADP-Glu necessary for starch synthesis.

**Fig. 5:**
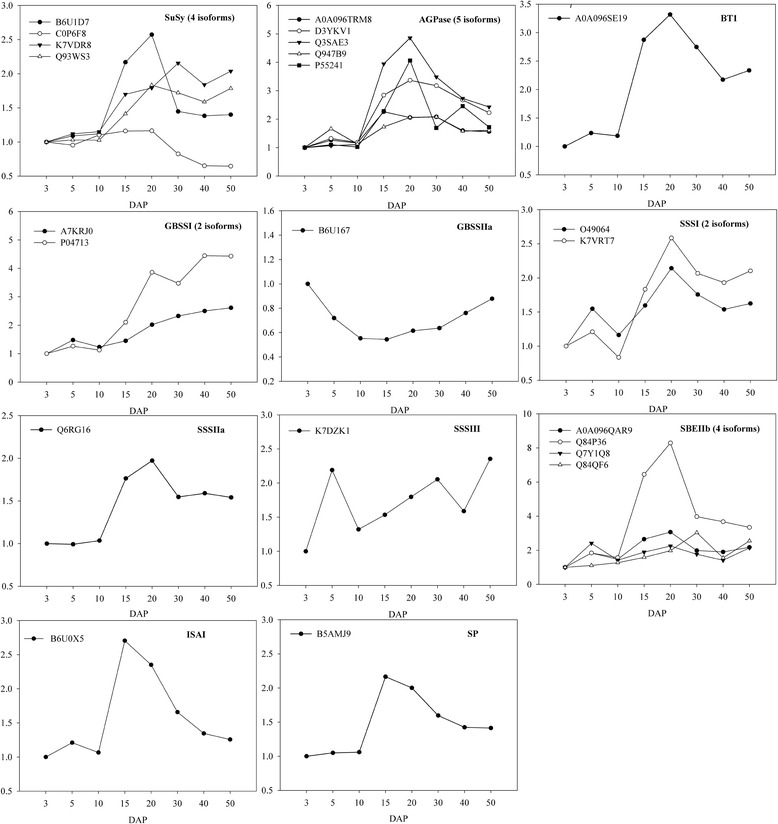
The expression levels of proteins involved in starch metabolism. The vertical axis shows the relative expression ratio to 3 DAP of each isoform at each developmental stage (DAP; horizontal axis)
2. SS and SBEIIb
  SS functions in the elongation of glucan chains through the action of granule-bound starch synthases (GBSSI and II) and soluble starch synthases (SSSI to IV) that are responsible for amylose and amylopectin synthesis, respectively [5]. Our study identified differential expression patterns of two isoforms of GBSSI, one of GBSSIIa, two of SSSI, one of SSSIIa, and one of SSSIII in developing grain (Fig. 5). GBSSIIa was down-regulated, whereas GBSSI was continuously up-regulated as starch accumulation increased (Fig. 5), supporting previous reports that GBSSI is dedicated to the synthesis of amylase [5, 49]. Previous studies have found that most SSS activity was dependent on SSSI and SSSIII products [50]. We found that SSSI peaked around 20 DAP, consistent with the dynamic changes of starch accumulation, whereas the abundance of SSSIII peaked at 6 and 50 DAP. Interestingly, SSSIIa was also identified as a DEP, and was found to show peak expression at 20 DAP, similar to the expression pattern of SSSI (Fig. 5). This result suggests that the contribution of SSSIIa to global SSS activity in the maize grain needs to be re-evaluated. Three types of starch branching enzyme (SBEIa, SBEIIa, and SBEIIb) are present in maize, and are involved in determining the branch density and branching pattern of amylopectin [51]. Four isoforms of SBEIIb were identified as DEPs (Fig. 5); three of the isoforms showed peak expression around 20 DAP, similar to the expression patterns of AGPase, BT1, SSSI, and SSSIIa. Together, these results suggested that synchronized expression and/or activity of AGPase, BT1, SBE, and SSS are essential for starch synthesis.
3. ISA and SP
  ISA is comprised of three types, namely ISAI, ISAII, and ISAIII, and is a starch debranching enzyme that hydrolyzes the α-1, 6-glucosic linkages of polyglucans. In contrast, SP catalyzes the reversible transfer of glucosyl units from G1P to the non-reducing end of an α-1, 4-linked glucan chain [5]. In this study, two DEPs were identified as ISAI and SP (Fig. 5), and both peaked in expression around 15 DAP. In rice grain, ISAs show their maximum accumulation at the grain filling stage [15], and they determined the amount of starch granules by affecting the initiation of starch granules [52]. In maize, ISAs were also required for normal starch granule growth [53]. Initially, it was thought that SP had a degradative rather than a biosynthetic function in starch metabolism; however, genetic analysis of rice grain mutants suggested a role for SP in facilitating the starch synthesis initiation process [54]. A recent study proposed that SP has a significant role in establishing the pool of linear short-chain malto-oligosaccharides that may serve as a primer for starch synthesis in rice grains [55]. Therefore, in maize grain, the co-expression of ISA and SP during rapid starch accumulation stage suggests that SP may have a role in the process of starch synthesis initiation.

### Protein expression characteristics during grain maturation stage

At the maturation stage (40–50 DAP), grains showed slow accumulation of storage materials but also began to undergo desiccation to finally acquire desiccation tolerance. Almost all storage related DEPs (16 of 21 proteins; e.g., globulins, legumins, alpha and gamma zeins) accumulated at their maximal levels at this stage (c2 and c3; Table 1). These proteins gradually degenerate during germination and act as a nitrogen source and carbon skeleton for seedling growth and development. The folding of nascent polypeptides into mature proteins is controlled by a number of molecular chaperones and protein-folding catalysts. Accordingly, a substantial proportion of DEPs involved in protein folding/modification (29 of 77 proteins; e.g., heat shock proteins and chaperones/chaperonins) were concurrently expressed with storage proteins (c2 and c3; Table 1); these proteins are good candidates for studying the folding and assembly of storage proteins in maize grain. Surprisingly, many DEPs related to proteolysis (23 of 60 proteins) also showed significant accumulation at this stage (c2 and c3; Table 1), and most of these were ubiquitin proteins (13 of 23 proteins). Ubiquitin is a highly conserved protein, and ubiquitination is a major modifier of signaling in all eukaryotes. The ubiquitin/26S proteasome pathway is the primary proteolysis mechanism for diverse cellular and developmental events [42, 43]. Therefore, the changes in expression abundance of ubiquitin proteins indicated that ubiquitination might play an important role during the maturation stage.

Interestingly, we also noticed four oleosins (B6SIZ2, B6SI42, B6TMT0 and P21641) and one steroleosin (B6UGU4) showed their highest accumulation levels at a late stage (Additional file 4). Both oleosins and steroleosins can be embedded in the monolayer membrane of phospholipids that surround oilbodies, the main lipid storage organelle in cereal crops [56, 57]. Oleosins are the major proteins preventing oilbody coalescence [56] and/or modulating the size of oilbodies [57]. During maize grain dehydration, oilbodies may coalesce due to cytoplasmic compression; thus, the accumulation of oleosins and steroleosins may have a role in the control of oilbody structure and lipid accumulation in maize grain.

During the maturation stage, another remarkable feature is that a large number of DEPs related to stress/defense (76 of 105 proteins) showed maximal accumulation (c2 and c3; Table 1); examples are late embryogenesis abundant (LEA) proteins, including one from LEA group 1 (K7VM99), three from LEA 14-A (B4F9K0, B4G1C1 and B6UH99), three from LEA group 3 (B6SID7, B6SJ28 and B6UI06), and four from LEA D-34 (A0A096TZ44, B6SN63, B6SNS4 and B6UH67) (Additional file 4). The increased expression of LEA proteins during the grain maturation stage has also been observed in rice [19] and wheat [31]. The presence and increased level of LEA proteins is correlated with desiccation tolerance [58, 59], and their expression is also induced in response to diverse abiotic or biotic stresses [59]. Therefore, the LEA proteins may be involved in protecting grain from serious dehydration at the late developmental stage. A recent proteomics study suggested that LEA proteins may also be an essential factor for maize grain viability [35]. Overall, these stress/defense-related proteins indicate the presence of a coordinated adverse response and defense mechanism during maize grain development, which could protect grain from adverse environments. More importantly, single or multiple proteins related to stress/defense might be of use as protein markers for the breeding of stress tolerant maize.

### ROS homeostasis regulation during grain development

At the early stages of maize grain development (4–14 DAP), oxygen levels are high and mitochondrial respiration is intense; at the late stages, grain primarily suffer from various stresses such as dehydration and hypoxia [60]. Under stress conditions, reactive oxygen species (ROS), such as superoxide radicals, hydrogen peroxide, and hydroxyl radicals, can be continuously generated [61]; these can damage cellular components but are also important for signaling in the regulation of many biological processes [62]. Cells have developed a wide range of antioxidant systems to maintain ROS homeostasis [61, 62]. In developing maize grain, 58 DEPs were identified as ROS related proteins (e.g., dehydroascorbate reductase, glutathione S-transferase, superoxide dismutase, and thioredoxin); these were involved in diverse antioxidant systems [61]. Further analysis showed that 14, 12, and 17 DEPs were at their maximal levels of accumulation in early, middle, and late stages, respectively. Fifteen DEPs showed significant accumulation at both early and late stages (Table 1; Additional file 4). The diverse expression patterns of these proteins suggested coordination of multiple complex antioxidant systems at different developmental stages. Among the identified DEPs were 1-cys-peroxiredoxin (1cys-Prx; A2SZW8) and zinc metallothionein class II (P43401), both of which showed maximum accumulation levels at the late stage (Additional file 4). Although 1cys-Prx was proposed to be involved in the maintenance of grain dormancy [63], other lines of evidence indicate that it has no significant correlation with dormancy but does confer higher resistance to oxidative stress [64] or can act as a sensor to inhibit grain germination under unfavorable conditions [65]. A recent study has further proposed that 1cys-Prx in grain may act as a molecular chaperone for protection of grain development under severe conditions [66]. The zinc metallothionein class II protein belongs to the metallothionein family, which might play important roles in maintaining essential metal homeostasis, detoxification of toxic metals, and protection against intercellular oxidative stress [67, 68]. In light of these functions, the high level of zinc metallothionein class II accumulation may be required for functions against intercellular oxidative stress and/or provide a means for storing Zn and other metals required for seedling growth after germination. The peak accumulation of zinc metallothionein class II proteins was similarly observed during the late stage of wheat grain development [69].

### Possible role for PPDK in starch synthesis and energy supply

The pyruvate orthophosphate dikinase (PPDK) catalyzes the reversible conversion of pyruvate (Pyr), ATP, and Pi into phosphoenolpyruvate (PEP), AMP, and PPi. It is a photosynthetic enzyme in the C4 cycle, but many proteomic studies have found that multiple isoforms of PPDK accumulate at high levels in the developing grain of cereals such as rice [15] and wheat [31], indicating that PPDK has an essential role in grain development. In maize, the genome has two loci encoding three types of PPDK proteins: *PPDKZM1* encode a C4-type chloroplastic PPDK1 and cytosolic PPDK1 by alternative splicing, and *PPDKZM2* contributes another cytosolic PPDK2 [70]. In this study, PPDK1 (B7ZYP6) and PPDK2 (K7UZT6) were identified as DEPs, and both grouped into cluster 1, displayed low expression levels during the early stage, increased significantly at 15–20 DAP, and decreased thereafter (Table 1; Additional file 4). In contrast, in rice grain, PPDK proteins are mostly expressed in the early stage rather than the grain filling stage [71]. The different expression patterns among species, as well as the cycle between PEP and Pyr and the PPi-ATP balance, might reflect the multiple functions of PPDK proteins during grain development [71]. However, their precise function (s) in maize grain development remains to be elucidated.

Based on our results, PPDK may function preferentially in the PEP to Pyr forming direction. Consistent with the PPDK expression pattern, most DEPs related to glycolysis (15 of 24 proteins) showed their highest expression levels at 15–20 DAP (c1; Table 1). The exception was pyruvate kinase (PK, B6TII5) which is an irreversible enzyme that converts PEP to Pyr; this enzyme showed continuous down-regulation during grain development (Additional file 4). These results suggest that proteins involved in active glycolysis participate in reactions leading to the increased production of PEP, whereas PEP is not efficiently converted to Pyr owing to the down regulation of PK. Meanwhile, most of the proteins involved in alcoholic fermentation (5 of 7 proteins), such as pyruvate decarboxylase, alcohol dehydrogenase, and those involved in the pyruvate dehydrogenase complex (3 of 5 proteins), were preferentially grouped into cluster 1 (Table 1; Additional file 4). This pattern of expression was comparable with that of PPDK and glycolysis. These results indicate that Pyr, as a reaction substrate, was a principal target for the active pyruvate dehydrogenase complex and alcoholic fermentation pathway. Therefore, as an additional complement pathway, PPDK may act to catalyze PEP to generate sufficient Pyr for the above two processes.

Importantly, Pyr formation may be beneficial to starch synthesis and energy supply at the grain filling stage. Our results showed that most starch synthesis related proteins including AGPase were grouped into cluster 1 (Table 1; Additional file 4), which was consistent with the PPDK expression pattern. As a key rate-limiting enzyme of starch synthesis, AGPase catalyzes a completely reversible reaction, and the direction of the reaction depends on the relative concentrations of PPi and ATP. Thus, the PPDK-dominated PEP to Pyr formation might reduce cytosolic PPi accumulation and push the reaction to ADP-Glu synthesis, which facilitates starch synthesis and accumulation. This proposal is supported by the findings of another study in rice grain in which mutations of the gene encoding PPDK showed the importance of the function of this enzyme in starch synthesis [72]. In addition, the cereal endosperm of species such as maize and rice is typically a hypoxic tissue in which ATP generation is inhibited by a decrease in internal oxygen concentration during grain development [60, 73]. Therefore, the PPDK-dominated PEP to Pyr formation may contribute to the energy supply by converting AMP to ATP and also by producing Pyr as a substrate for the active alcoholic fermentation pathway (see above). The latter pathway generates ATP without the consumption of oxygen [74], and helps to maintain the appropriate ATP level for starch synthesis under low oxygen tension. A further clue to PPDK function is provided by the fact that its expression is enhanced under low-oxygen stress in rice roots [75]. Together, these lines of evidence indicate that PPDK may function preferentially in the PEP to Pyr forming direction, and thereby reduce cytosolic PPi accumulation and increase ATP content, to finally facilitate starch synthesis and energy supply at the grain filling stage.

### 14-3-3 proteins may perform an important role during grain development

In this study, three isoforms of 14-3-3 proteins (B4FRG1, B6SZB9, and B6T7L9) were identified as DEPs. Interestingly, all three showed significant accumulation at the early stage (3–10 DAP), and then decreased to low levels until grain maturity (c1; Table 1). Similar expression patterns have also been observed in other species, such as castor [22] and rice [15], suggesting a possible role in grain development. Several studies have reported that 14-3-3 proteins are involved in various cellular physiological processes, such as cell signal transduction, cell cycle regulation, nitrogen and carbon assimilation, and defense mechanisms [76, 77]. In barley and maize grain, 14-3-3 proteins might interact with starch synthesis related enzymes, such as ADPase, GBSS, SBE, and ISA [78, 79], indicating that they might be involved in the regulation of starch synthesis. Interestingly, in *Arabidopsis* leaves, reduction or over-expression of 14-3-3 proteins is correlated with dramatic increases or decreases, respectively, in starch content [80, 81]. In addition, proteomic and western blotting analyses of rice grain showed that 14-3-3 proteins display a lower level of expression in grains with high starch content than in grain with low starch content [82]. Therefore, it has been suggested that high expression of 14-3-3 proteins may decrease the activity of enzymes related to starch synthesis, and may consequently be detrimental to starch formation and accumulation [80, 82]. Consistent with this hypothesis, we found here that the highest level of expression of 14-3-3 proteins occurred at the early stage, and that this level fell dramatically after 10 DAP when starch synthesis enzymes began to be up-regulated. Nonetheless, the underlying mechanism of 14-3-3 proteins in regulating starch synthesis still remains to be elucidated; determination of the role of these proteins may be of value for improving starch productivity in crop plants.

## Conclusions

We explored the dynamic changes in protein expression during eight sequential developmental stages from 3 to 50 DAP in maize grain. Applying iTRAQ technology, 4751 proteins were identified and 1235 were classified as DEPs during grain development, reflecting the fact that iTRAQ-based quantitative proteome analysis is a powerful technique for describing complex metabolic processes. Our results indicated that coordination of metabolism and cellular processes is associated with different developmental stages in grain; for example, the DEPs involved in cell growth/division are down-regulated after the early stage, whereas those related to starch biosynthesis and defense/stress are significantly up-regulated at the middle and late stages, respectively. We also demonstrate coordination of a multiplicity of proteins in the antioxidant system at different developmental stages, which is essential for maintenance of ROS homeostasis. In addition, some DEPs, such as zinc metallothionein class II, PPDK, and 14-3-3 proteins, undergo major changes in expression at specific developmental stages, suggesting their important roles in maize grain development. These results provide novel clues for the further understanding of the molecular mechanisms influencing maize grain yield and quality.

## Methods

### Plant material and sampling

The elite Chinese maize cultivar Denghai 661 (DH351/DH372) was used in this study. The seed was obtained from Shandong Denghai Seeds Co., Ltd. (Laizhou, China). Plants were grown during the maize growing season at the experimental farm of Shandong Agricultural University, Taian (36°10′E, 117°04′N), China. Plants flowering on the same day were tagged and artificially self-pollinated. Nine ears were collected at each stage of 3, 5, 10, 15, 20, 30, 40, and 50 DAP. In order to increase the uniformity of the material, fertilized grains from the middle part of each ear were sampled. For each stage, three samples were prepared by mixing an equal number of grains from three cobs; the samples were stored immediately at −80 °C until protein extraction. Fresh weight and dry weight were measured at each grain stage. Grains of 10–50 DAP and 3–30 DAP were collected for the determination of total starch content and number of endosperm cells, respectively, as described previously [83].

### Protein extraction

Grain samples were ground into fine powder in liquid nitrogen using a mortar and pestle; the powder was suspended in a 10-fold volume of precooled acetone (−20 °C) containing 10 % (v/v) trichloroacetic acid (TCA). The homogenate was then precipitated for 2 h at −20 °C after thorough mixing. The homogenate was then centrifuged for 30 min at 20,000 *g* at 4 °C, and the supernatant was carefully removed; the pellet was rinsed three times with cold acetone, left at −20 °C for 30 min, and then centrifuged at 20,000 *g* for 30 min at 4 °C. The resulting pellets was dissolved in lysis buffer containing 8 M urea, 30 mM HEPES, 1 mM polyvinylpolypyrrolidone (PMSF), 2 mM EDTA, and 10 mM dithiothreitol (DTT) and then sonicated for 5 min. The dissolved protein extract was centrifuged at 20,000 *g* for 30 min at 4 °C, the supernatant was collected and reduced with 10 mM DTT at 56 °C for 1 h, and then alkylated with 55 mM iodoacetamide (IAM) for 1 h in the dark. The mixture was precipitated using a 5-fold volume of cold acetone at −20 °C for 3 h, followed by centrifugation at 20,000 *g* for 30 min. The resulting pellet was dissolved in 0.5 M triethylammonium bicarbonate (TEAB) buffer with 0.1 % SDS, sonicated for 5 min, and centrifuged at 20,000 *g* for 30 min. The supernatant was used for liquid digestion, and the protein concentration was determined using the Bradford assay (Bio-Rad, Hercules, CA, USA) with BSA as a standard.

### In solution digestion and iTRAQ labeling

For each sample, 3.3 μL trypsin (1 μg/μL) (Promega, Madison, WI, USA) was added to 100 μg of protein in TEAB buffer and the proteins were digested at 37 °C for 24 h. A fresh aliquot of trypsin (1 μL) was added, and the sample was digested again for 12 h. The precipitate was dissolved in 30 μL 0.5 M TEAB and mixed with 70 μL isopropanol. Then, the digested peptides were labeled with iTRAQ reagents (AB SCIEX, Framingham, MA, USA) according to the manufacturer’s instructions. The grain samples obtained from 3, 5, 10, 15, 20, 30, 40, and 50 DAP were labeled with iTRAQ reagents 113, 114, 115, 116, 117, 118, 119, and 121, respectively. Three independent biological experiments were performed.

### SCX and LC-MS/MS

The pooled peptides were dissolved in strong cation exchange (SCX) buffer A (10 mM potassium phosphate monobasic (KH_2_PO_4_) in 25 % acetonitrile, pH 2.8). The mixture was adjusted to pH 3 using phosphoric acid, and then fractionated using a high-performance liquid chromatography (HPLC) system (Shimadzu, Kyoto, Japan) equipped with a silica-based SCX column (250 × 4.6 mm, 5 μm, 100 Å, Phenomenex, Torrance, CA, USA). In total, 36 fractions were collected at a flow rate of 1 mL/min with buffer B (10 mM KH_2_PO_4_ and 2 M potassium chloride (KCl) in 25 % acetonitrile, pH 2.8) with the following gradient: 0 % for 45 min, 0–5 % for 1 min, 5–30 % for 20 min, 30–50 % for 5 min and maintained for 5 min, and 50–100 % for 5 min, and maintained for 10 min. The fractions were desalted with a strata-X 33 μm PolyRevStage SPE (Phenomenex) following the manufacturer’s instructions and lyophilized in a centrifugal speed vacuum concentrator. Then, 30 μL of 0.1 % formic acid was added to each dried fraction tube, and 0.1 μL of the re-dissolved solution was spotted on the target well of an Anchor-chip plate for MALDI-TOF testing. After the MALDI-TOF (Bruker Daltonics, Germany) test, the 36 fractions were combined into 16 final fractions according to the peak area.

The mass spectrometry analysis was performed on a Dionex Ultimate 3000 Nano LC system connected to a Q-Exactive mass spectrometer (Thermo Fisher Scientific, MA, USA). The peptide mixtures were loaded onto a Acclaim PePmap C18-reversed phase column (75 μm × 2 cm, 3 μm, 100 Å, Thermo Scientific) and separated with a reversed phase C18 column (75 μm × 10 cm, 5 μm, 300 Å, Agela Technologies) using a gradient of 5–80 % (v/v) acetonitrile in 0.1 % formic acid over 45 min at a flow rate of 300 nL/ min. Solvent A was 0.1 % formic acid in water. A full mass spectrometry (MS) scan (350–2000 m/z) was acquired in the positive ion mode at a resolution of 70,000 (at 200 m/z), an AGC target value of 3–6, a maximum ion accumulation time of 50 ms, number of scan ranges of 1, and dynamic exclusion of 15 s. Information on peptides and peptide fragments m/z were obtained using the following conditions: 20 fragment files were collected after every full scan (MS2 scan), higher collision energy dissociation (HCD) fragmentation, an isolation window of 2 m/z, full scan at a resolution of 17,500 (at 200 m/z), micro-scans of 1, a maximum ion accumulation time of 100 ms, normalized collision energy of 28 eV, and an under-fill ratio of 1 %.

### Data analysis

For protein identification, the MS raw files were processed with Proteome Discoverer 1.3 (Thermo Fisher Scientific) and searched with in-house MASCOT software 2.3.01 (Matrix Science, London, UK). The acquired MS/MS spectra were automatically searched against a UniProt-*Zeamays* protein database (86,922 sequences in December 2014). The search parameters were as follows: trypsin was chosen as the enzyme with one missed cleavage allowed; fixed modifications of carbamidomethylation of cysteine residues; iTRAQ 8-plex modification of the N terminus, K and Y, Gln → Pyro-Glu of the N terminus and oxidation of methionine were set as variable modifications; peptide tolerance was set at 15 ppm; and MS/MS tolerance was set at 20 mmu. At least one unique peptide with a false discovery rate (FDR) ≤1 % was required for protein identification and quantification data analysis.

Two criteria were used for the quantitation of the identified proteins: 1) the median protein ratio was chosen; 2) the minimum precursor charge was set to 2 and only unique peptides were used for quantitation. The labeled samples obtained at 3 DAP were used as a reference (REF) based on the weighted average of the intensity of report ions in each identified peptide. To indicate the abundance of a protein at each stage, the relative protein ratios of samples of each stage against 3 DAP were calculated as the median of all peptides belonging to the assigned sample (3 d/REF, 5 d/REF, 10 d/REF, 15 d/REF, 20 d/REF, 30 d/REF, 40 d/REF, and 50 d/REF). For analysis of DEPs during grain development, only proteins with quantitative information from at least two biological replicates were used. The average of three biological replicates was used to indicate final protein abundance at each stage, and proteins showing average protein abundance that changed significantly by more than 1.5-fold in different stages (ANOVA test, p ≤ 0.05) were defined as DEPs. The K-means clustering analysis of the log-transformed fold-change expression values for the DEPs was conducted with Cluster 3.0 software (http://bonsai.hgc.jp/~mdehoon/software/cluster/software.htm) using similarity metric and Euclidean distances. The number of clusters was set as 5 and the result was visualized using the associated Java TreeView 1.1.1 software.

## Additional files

Additional file 1: Total proteins identified in maize grains (XLSX 745 kb)
Additional file 2: Expression profile data for quantified proteins (XLSX 1136 kb)
Additional file 3: The homologs of uncharacterized proteins in maize grains (XLSX 48 kb)
Additional file 4: The DEPs were allocated to functional categories and cluster membership (XLSX 166 kb)

## Abbreviations

1cys-Prx: 1-cys-peroxiredoxin
2-DE: two-dimensional electrophoresis
6PGDH: 6-phosphogluconate dehydrogenase
ADP-Glu: ADP-glucose
AGPase: ADP-glucose pyrophosophorylase
BT1: ADP-glucose brittle-1 transporter
DAP: day after pollination
DEPs: differentially expressed proteins
G1P: glucose-1-phosphate
G6PDH: glucose-6-phosphate 1-dehydrogenase
GBSS: granule-bound starch synthase
ISA: isoamylase
iTRAQ: isobaric tag for relativeand absolute quantitation
LEA: late embryogenesis abundant
PEP: phosphoenolpyruvate
PPDK: pyruvate orthophosphate dikinase
PPP: pentose phosphate pathway
Pyr: pyruvate
RNA-seq: RNA sequencing
ROS: reactive oxygen species
SBE: starch branching enzyme
SSS: soluble starch synthase
SuSy: sucrose synthase
TCA: tricarboxylic acid

## References

[1] Prioul JL, Méchin V, Lessard P, Thévenot C, Grimmer M, Chateau-Joubert S (2008). A joint transcriptomic, proteomic and metabolic analysis of maize endosperm development and starch filling. Plant Biotechnol J.

[2] Olsen OA (2001). Endosperm development. cellularization and cell fate specification. Annu Rev Plant Biol.

[3] Sabelli PA, Larkins BA (2009). The development of endosperm in grasses. Plant Physiol.

[4] Nelson O, Pan D (1995). Starch synthesis in maize endosperms. Annu Rev Plant Biol.

[5] Jeon J, Ryoo N, Hahn T, Walia H, Nakamura Y (2010). Starch biosynthesis in cereal endosperm. Plant Physiol Biochem.

[6] James MG, Denyer K, Myers AM (2003). Starch synthesis in the cereal endosperm. Curr Opin Plant Biol.

[7] Jin X, Fu Z, Ding D, Li W, Liu Z, Tang J (2013). Proteomic identification of genes associated with maize grain-filling rate. PLoS One.

[8] Méchin V, Thévenot C, Le Guilloux M, Prioul JL, Damerval C (2007). Developmental analysis of maize endosperm proteome suggests a pivotal role for pyruvate orthophosphate dikinase. Plant Physiol.

[9] Li G, Wang D, Yang R, Logan K, Chen H, Zhang S (2014). Temporal patterns of gene expression in developing maize endosperm identified through transcriptome sequencing. Proc Natl Acad Sci U S A.

[10] Chen J, Zeng B, Zhang M, Xie S, Wang G, Hauck A (2014). Dynamic transcriptome landscape of maize embryo and endosperm development. Plant Physiol.

[11] Verza NC, e Silva TR, Neto GC, Nogueira FT, Fisch PH, de Rosa VE (2005). Endosperm-preferred expression of maize genes as revealed by transcriptome-wide analysis of expressed sequence tags. Plant Mol Biol.

[12] Sekhon RS, Lin H, Childs KL, Hansey CN, Buell CR, de Leon N (2011). Genome-wide atlas of transcription during maize development. Plant J.

[13] Teoh KT, Requesens DV, Devaiah SP, Johnson D, Huang X, Howard JA (2013). Transcriptome analysis of embryo maturation in maize. BMC Plant Biol.

[14] Walley JW, Shen Z, Sartor R, Wu KJ, Osborn J, Smith LG (2013). Reconstruction of protein networks from an atlas of maize seed proteotypes. Proc Natl Acad Sci U S A.

[15] Xu SB, Li T, Deng ZY, Chong K, Xue Y, Wang T (2008). Dynamic proteomic analysis reveals a switch between central carbon metabolism and alcoholic fermentation in rice filling grains. Plant Physiol.

[16] Hu G, Koh J, Yoo MJ, Grupp K, Chen S, Wendel JF (2013). Proteomic profiling of developing cotton fibers from wild and domesticated *Gossypium barbadense*. New Phytol.

[17] Hajduch M, Hearne LB, Miernyk JA, Casteel JE, Joshi T, Agrawal GK (2010). Systems analysis of seed filling in Arabidopsis: using general linear modeling to assess concordance of transcript and protein expression. Plant Physiol.

[18] Tasleem-Tahir A, Nadaud I, Girousse C, Martre P, Marion D, Branlard G (2011). Proteomic analysis of peripheral layers during wheat (*Triticum aestivum* L.) grain development. Proteomics.

[19] Xu H, Zhang W, Gao Y, Zhao Y, Guo L, Wang J (2012). Proteomic analysis of embryo development in rice (*Oryza sativa*). Planta.

[20] Dupont FM (2008). Metabolic pathways of the wheat (*Triticum aestivum*) endosperm amyloplast revealed by proteomics. BMC Plant Biol.

[21] Finnie C, Melchior S, Roepstorff P, Svensson B (2002). Proteome analysis of grain filling and seed maturation in barley. Plant Physiol.

[22] Houston NL, Hajduch M, Thelen JJ (2009). Quantitative proteomics of seed filling in castor: comparison with soybean and rapeseed reveals differences between photosynthetic and nonphotosynthetic seed metabolism. Plant Physiol.

[23] Gallardo K, Le Signor C, Vandekerckhove J, Thompson RD, Burstin J (2003). Proteomics of *Medicago truncatula* seed development establishes the time frame of diverse metabolic processes related to reserve accumulation. Plant Physiol.

[24] Hajduch M, Ganapathy A, Stein JW, Thelen JJ (2005). A systematic proteomic study of seed filling in soybean. Establishment of high-resolution two-dimensional reference maps, expression profiles, and an interactive proteome database. Plant Physiol.

[25] Agrawal GK, Hajduch M, Graham K, Thelen JJ (2008). In-depth investigation of the soybean seed-filling proteome and comparison with a parallel study of rapeseed. Plant Physiol.

[26] Lilley KS, Razzaq A, Dupree P (2002). Two-dimensional gel electrophoresis: recent advances in sample preparation, detection and quantitation. Curr Opin Chem Biol.

[27] Wu WW, Wang G, Baek SJ, Shen RF (2006). Comparative study of three proteomic quantitative methods, DIGE, cICAT, and iTRAQ, using 2D gel-or LC-MALDI TOF/TOF. J Proteome Res.

[28] Karp NA, Huber W, Sadowski PG, Charles PD, Hseter SV, Lilley KS (2010). Addressing accuracy and precision issues in iTRAQ quantitation. Mol Cell Proteomics.

[29] Schulze WX, Usadel B (2010). Quantitation in mass-spectrometry-based proteomics. Annu Rev Plant Biol.

[30] Zi J, Zhang J, Wang Q, Zhou B, Zhong J, Zhang C (2013). Stress responsive proteins are actively regulated during rice (*Oryza sativa*) embryogenesis as indicated by quantitative proteomics analysis. PLoS One.

[31] Ma C, Zhou J, Chen G, Bian Y, Lv D, Li X (2014). iTRAQ-based quantitative proteome and phosphoprotein characterization reveals the central metabolism changes involved in wheat grain development. BMC Genomics.

[32] Méchin V, Balliau T, Chateau-Joubert S, Davanture M, Langella O, Negroni L (2004). A two dimensional proteome map of maize endosperm. Phytochemistry.

[33] Xin X, Lin XH, Zhou YC, Chen XL, Liu X, Lu XX (2011). Proteome analysis of maize seeds: the effect of artificial ageing. Physiol Plant.

[34] Huang H, Møller IM, Song SQ (2012). Proteomics of desiccation tolerance during development and germination of maize embryos. J Proteomics.

[35] Wu X, Liu H, Wang W, Chen S, Hu X, Li C (2011). Proteomic analysis of seed viability in maize. Acta Physiol Plant.

[36] Mayer U, Jurgens G (2002). Microtubule cytoskeleton a track record. Curr Opin Plant Biol.

[37] Herrera I, De La Paz Sánchez M, Molina J, Plasencia J, Vázquez-Ramos JM (2000). Proliferating cell nuclear antigen expression in maize grain development and germination: regulation by phytohormones and its association with putative cell cycle proteins. Physiol Plant.

[38] Guillaumie S, San-Clemente H, Deswarte C, Martinez Y, Lapierre C, Murigneux A (2007). MAIZEWALL. Database and developmental gene expression profiling of cell wall biosynthesis and assembly in maize. Plant Physiol.

[39] Martin SW, Glover BJ, Davies JM (2005). Lipid microdomains-plant membranes get organized. Trends Plant Sci.

[40] Bruhn H (2005). A short guided tour through functional and structural features of saposin-like proteins. Biochem J.

[41] Cochrane CG, Revak SD (1991). Pulmonary surfactant protein B (SP-B): structure-function relationships. Science.

[42] Moon J, Parry G, Estelle M (2004). The ubiquitin-proteasome pathway and plant development. Plant Cell.

[43] Vierstra RD (2009). The ubiquitin-26S proteasome system at the nexus of plant biology. Nat Rev Mol Cell Biol.

[44] Kruger NJ, von Schaewen A (2003). The oxidative pentose phosphate pathway: structure and organisation. Curr Opin Plant Biol.

[45] Horecker BL (2002). The pentose phosphate pathway. J Biol Chem.

[46] Wang F, Sanz A, Brenner ML, Smith A (1993). Sucrose synthase, starch accumulation, and tomato fruit sink strength. Plant Physiol.

[47] Shannon JC, Pien FM, Cao H, Liu KC (1998). Brittle-1, an adenylate translocator, facilitates transfer of extraplastidial synthesized ADP-glucose into amyloplasts of maize endosperms. Plant Physiol.

[48] Bowsher CG, Scrase-Field EF, Esposito S, Emes MJ, Tetlow IJ (2007). Characterization of ADP-glucose transport across the cereal endosperm amyloplast envelope. J Exp Bot.

[49] Shure M, Wessler S, Fedoroff N (1983). Molecular identification and isolation of the *waxy* locus in maize. Cell.

[50] Cao H, Imparl-Radosevich J, Guan H, Keeling PL, James MG, Myers AM (1999). Identification of the soluble starch synthase activities of maize endosperm. Plant Physiol.

[51] Yao Y, Thompson DB, Guiltinan MJ (2004). Maize starch-branching enzyme isoforms and amylopectin structure. In the absence of starch-branching enzyme IIb, the further absence of starch-branching enzyme Ia leads to increased branching. Plant Physiol.

[52] Kawagoe Y, Kubo A, Satoh H, Takaiwa F, Nakamura Y (2005). Roles of isoamylase and ADP-glucose pyrophosphorylase in starch granule synthesis in rice endosperm. Plant J.

[53] Kubo A, Colleoni C, Dinges JR, Lin QH, Lappe RR, Rivenbark JG (2010). Functions of heteromeric and homomeric isoamylase-type starch-debranching enzymes in developing maize endosperm. Plant Physiol.

[54] Satoh H, Shibahara K, Tokunaga T, Nishi A, Tasaki M, Hwang SK (2008). Mutation of the plastidial α-glucan phosphorylase gene in rice affects the synthesis and structure of starch in the endosperm. Plant Cell.

[55] Hwang SK, Nishi A, Satoh H, Okita TW (2010). Rice endosperm-specific plastidial α-glucan phosphorylase is important for synthesis of short-chain malto-oligosaccharides. Arch Biochem Biophys.

[56] Tzen JT, Huang AH (1992). Surface structure and properties of plant seed oil bodies. J Cell Biol.

[57] Siloto RM, Findlay K, Lopez-Villalobos A, Yeung EC, Nykiforuk CL, Moloney MM (2006). The accumulation of oleosins determines the size of seed oilbodies in Arabidopsis. Plant Cell.

[58] Amara I, Odena A, Oliveira E, Moreno A, Masmoudi K, Pagès M (2012). Insights into maize LEA proteins: from proteomics to functional approaches. Plant Cell Physiol.

[59] Battaglia M, Olvera-Carrillo Y, Garciarrubio A, Campos F, Covarrubias AA (2008). The enigmatic LEA proteins and other hydrophilins. Plant Physiol.

[60] Rolletschek H, Koch K, Wobus U, Borisjuk L (2005). Positional cues for the starch/lipid balance in maize kernels and resource partitioning to the embryo. Plant J.

[61] Mittler R, Vanderauwera S, Gollery M, Van Breusegem F (2004). Reactive oxygen gene network of plants. Trends Plant Sci.

[62] Foyer CH, Noctor G (2005). Redox homeostasis and antioxidant signaling: a metabolic interface between stress perception and physiological responses. Plant Cell.

[63] Stacy RA, Nordeng TW, Culianez-Marcia FA, Aalen RB (1999). The dormancy-related peroxiredoxin antioxidant, PER1, is localized to the nucleus of barley embryo and aleurone cells. Plant J.

[64] Lee KO, Jang HH, Jung BG, Chi YH, Lee JY, Choi YO (2000). Rice 1Cys-peroxiredoxin over-expressed in transgenic tobacco does not maintain dormancy but enhances antioxidant activity. FEBS Lett.

[65] Haslekås C, Viken MK, Grini PE, Nygaard V, Nordgard SH, Meza TJ (2003). Seed 1-cysteine peroxiredoxin antioxidants are not involved in dormancy, but contribute to inhibition of germination during stress. Plant Physiol.

[66] Kim SY, Paeng SK, Nawkar GM, Maibam P, Lee ES, Kim KS (2011). The 1-Cys peroxiredoxin, a regulator of seed dormancy, functions as a molecular chaperone under oxidative stress conditions. Plant Sci.

[67] Klaassen CD, Liu J, Choudhuri S (1999). Metallothionein: an intracellular protein to protect against cadmium toxicity. Annu Rev Pharmacol.

[68] Guo WJ, Bundithya W, Goldsbrough PB (2003). Characterization of the Arabidopsis metallothionein gene family: tissue-specific expression and induction during senescence and in response to copper. New Phytol.

[69] Kawashima I, Kennedy TD, Chino M, Lane BG (1992). Wheat Ec metallothionein genes: like mammalian Zn^2+^ metallothionein genes are conspicuously expressed during embryogenesis. Eur J Biochem.

[70] Sheen J (1991). Molecular mechanisms underlying the differential expression of maize pyruvate, orthophosphate dikinase genes. Plant Cell.

[71] Chastain CJ, Heck JW, Colquhoum TA, Voge DG, Gu XY (2006). Post-translational regulation of pyruvate orthophosphate dikinase in developing rice (*Oryza sativa*) seeds. Planta.

[72] Kang HG, Park S, Matsuoka M, An G (2005). White-core endosperm floury endosperm-4 in rice is generated by knockout mutations in the C-type pyruvate orthophosphate dikinase gene (*OsPPDKB*). Plant J.

[73] Xu SB, Yu HT, Yan LF, Wang T (2010). Integrated proteomic and cytological study of rice endosperms at the storage phase. J Proteome Res.

[74] Geigenberger P (2003). Response of plant metabolism to too little oxygen. Curr Opin Plant Biol.

[75] Moons A, Valcke R, Van M (1998). Low-oxygen stress and water deficit induce cytosolic pyruvate orthophosphate dikinase (PPDK) expression in roots of rice, a C3 plant. Plant J.

[76] Chevalier D, Morris ER, Walker JC (2009). 14-3-3 and FHA domains mediate phosphoprotein interactions. Annu Rev Plant Biol.

[77] Oecking C, Jaspert N (2009). Plant 14-3-3 proteins catch up with their mammalian orthologs. Curr Opin Plant Biol.

[78] Alexander RD, Morris PC (2006). A proteomic analysis of 14-3-3 binding proteins from developing barley grains. Proteomics.

[79] Dou Y, Liu X, Yin Y, Han S, Lu Y, Liu Y (2015). Affinity chromatography revealed insights into unique functionality of two 14-3-3 protein species in developing maize kernels. J Proteomics.

[80] Sehnke PC, Chung HJ, Wu K, Ferl RJ (2001). Regulation of starch accumulation by granule-associated plant 14-3-3 proteins. Proc Natl Acad Sci U S A.

[81] Diaz C, Kusano M, Sulpice R, Araki M, Redestig H, Saito K (2011). Determining novel functions of Arabidopsis 14-3-3 proteins in central metabolic processes. BMC Syst Biol.

[82] Zhang Z, Zhao H, Tang J, Li Z, Li Z, Chen D (2014). A proteomic study on molecular mechanism of poor grain-filling of rice (*Oryza sativa* L.) inferior spikelets. PloS One.

[83] Liu YE, Liu P, Dong ST, Zhang JW (2010). Hormonal changes caused by the xenia effect during grain filling of normal corn and high-oil corn crosses. Crop Sci.

